# How Much Is That in Dog Years? The Advent of Canine Population Genomics

**DOI:** 10.1371/journal.pgen.1004093

**Published:** 2014-01-16

**Authors:** Greger Larson, Daniel G. Bradley

**Affiliations:** 1 Durham Evolution and Ancient DNA, Department of Archaeology, University of Durham, Durham, United Kingdom; 2 Smurfit Institute of Genetics, Trinity College Dublin, Dublin, Ireland; Uppsala University, Sweden

Imprecision in determining when and where dogs were first domesticated has vexed geneticists for the past 20 years and archaeologists for many decades longer. This has been particularly frustrating since dogs were certainly the first domesticated taxa, so understanding when and where our relationship with dogs began is crucial to comprehending the transition of humans from hunter-gatherers to farmers.

Genetic efforts to query the time and place of dog domestication have moved from mtDNA phylogeography through several generations of autosomal marker analysis and now enter an exciting new phase: the interrogation of whole genome sequences. Freedman et al. [1] present one of two recent papers (including Wang et al. [2]) that generate and analyze multiple genomes of dogs and wolves. However, the approaches, sampling, and conclusions differ significantly between the two papers.

## Dating the Divergence: Dogged by Mutation Rate Estimate Variation

Establishing the precise geography and timing of dog domestication using archaeology has been difficult for several reasons. Firstly, because wolves were once distributed across the entire Northern Hemisphere, zooarchaeologists have not been able to establish the wild or domestic status of fossil canid remains based solely on geographic location; thus cranial and dental characters have had to be used to differentiate domestic dogs from wild wolves. Despite uncertainty regarding natural morphological variation, the earliest appearance of dogs has been placed at about 15,000 years ago in Europe and the Far East. More recently, claims have been made that canid remains dated to about 30,000 years ago in Belgium, Ukraine, and Russia are either of early dogs or failed efforts at dog domestication; though some archaeologists remain unconvinced.

**Figure 1:**
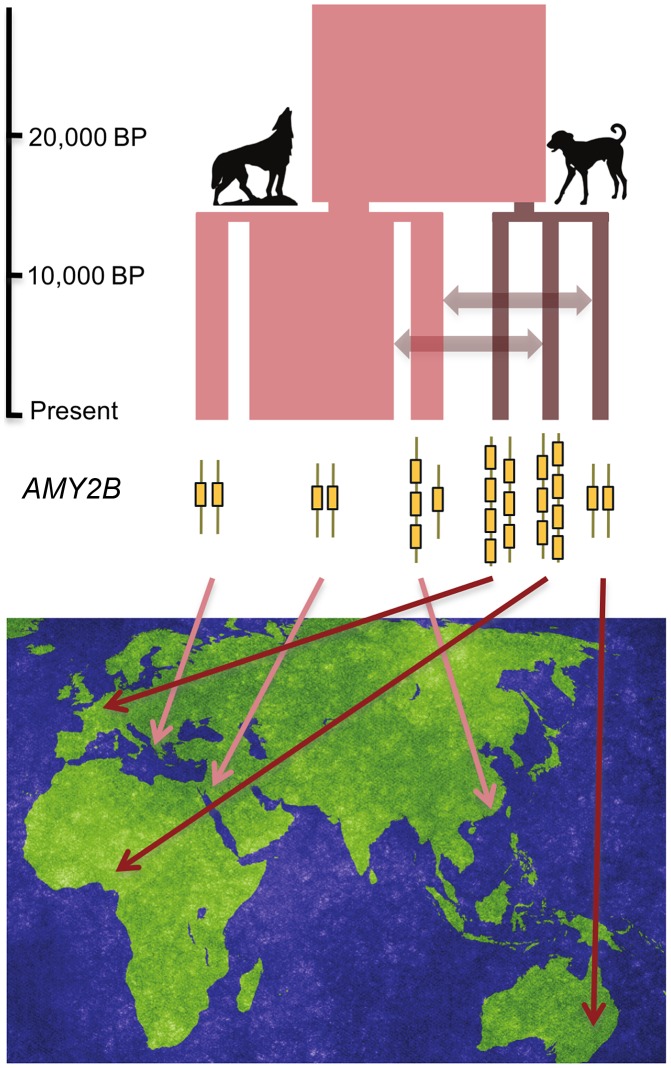
Summary of the demographic model and sampling from Freedman et al. [1]. Their critical inclusion of data from the Australian Dingo illustrates that high copy number in *AMY2B* is not a basal trait in dogs. The reciprocal monophyly of wild and domestic suggests that, despite the geographical diversity of sampling, descendants of the wolf population that contributed to dog domestication are not represented and may only be accessible using ancient DNA.

Geneticists first entered the fray in 1997 when, using mitochondrial control region fragments of dogs and wolves, Vila et al. [3] concluded that the two lineages diverged 135,000 years ago. Subsequent genetic studies have produced a wide range of estimates, often with large confidence intervals, and despite the generation of ever-larger data sets, date ranges have not yet begun to converge. For example, despite the fact that both Wang and Freedman generated high-coverage complete genomes from multiple distantly related dogs and wolves, they reach different conclusions about the date and population effects. Wang et al. [2] concluded that dogs and wolves diverged 32,000 years ago and that the domestication bottleneck was relatively mild, while Freedman et al. [1], in their closely argued analysis, placed the wolf-dog bifurcation at 11,000–16,000 years ago and concluded that the domestication process resulted in a 16-fold reduction in population size (Figure 1).

The primary reason for this disparity is reliance on molecular evolutionary rates that differ by an order of magnitude. As Freedman et al. [1] point out, little is known about the dog-specific mutation rate. By incorporating the entire range of published estimates, they demonstrate that the mutation rate is “the dominant source of uncertainty in dating the origin of dogs.” The use of the entire range of rates therefore results in a credible interval of the origin of dogs from 9,000–34,000 years ago, certainly in greater agreement with the archaeological estimates, but still lacking precision.

## Did Dogs Originate Before or After Agriculture?

Though they may differ on whether the recently described 30,000-year-old canids were dogs, all zooarchaeologists support the contention that dogs were not only the first domestic animal, but that the appearance of dogs significantly predates the origins of domestic plants and early agriculture. They base this conclusion on the fact that the earliest dog bones found across the Old World from Europe to the Near East to the Kamchatka Peninsula have been reliably dated to several millennia prior to the first archaeological appearance of domesticated crops in the Near East and East Asia [4].

A recent study of pooled resequenced whole genomes revealed that dogs possessed a seven-fold increase in the copy number of the *AMY2B* locus, a gene involved in amylase activity crucial to the digestion of starches. Based upon this observation, Axelsson et al. [5] concluded that the shift away from a more carnivorous diet was central and that the “development of agriculture catalysed the domestication of dogs.” In other words, the genomic evidence for copy number variation in dietary genes between dogs and wolves suggested that the archaeologists were wrong, and that dogs were domesticated not before, but after the origin of agriculture.

Freedman et al. [1] investigated this locus in their study and found not only that the *AMY2B* copy number increase was not fixed across all dogs (their Dingo possessed only two copies while the Saluki had 29), but also that the observed variation was polymorphic in nearly half of 20 wolves under investigation. These results suggest a more complex pattern of amylase copy number variation in dogs and wolves that reflects our long-standing relationship with dogs, but may not have resulted during early domestication.

## Where Dogs Were Domesticated

Given the broad geographical range over which early dog remains have been discovered, archaeologists have been generally content to embrace the ambiguity of the zooarchaeological record and accept that there has not been sufficient evidence to support one or several geographic centers of dog domestication.

Many genetic studies have not been as reticent. For instance, though an early mitochondrial study concluded that dogs were domesticated just once in East Asia [4], a subsequent analysis of African village dogs [6] cast doubt on this claim. A more recent study [7] using >48,000 single-nucleotide polymorphisms in wolves and dogs concluded that East Asian and Near Eastern wolf populations both contributed DNA to modern dog breeds. Though studies of nuclear markers have suggested diverse geographic origins for dogs, several authors continue to insist that all dogs descend from a single East Asian wolf population.

One reason for these discrepancies is likely to be the sustained admixture between different dog and wolf populations across the Old and New Worlds over at least the last 10,000 years. This has blurred the genetic signatures and confounded efforts at pinpointing the origins of dogs [7]. Another more intriguing reason stems from Freedman et al.'s conclusion that dog and wolf lineages are reciprocally monophyletic, suggesting that none of the modern wolf populations are related to the wolves that were first domesticated. In other words, the extinction of the wolves that were the direct ancestors of dogs has muddied efforts to pinpoint the time and place of dog domestication.

The sequencing of multiple complete, high-quality genomes of dogs and wolves is a significant step forward in the genetic hunt for the origins of our earliest domestic animal. The trick now is to extend the application of these methods to ancient remains: in effect, merging the materials and methods of both archaeology and genetics. By combining the expertise of both disciplines, not only might the extinct population of ancestral wolves be identified, but we will gain an enormous insight into the timing, location, and admixture patterns of dogs and wolves, thus revealing the complex origins of our first and best friend.
